# The combination of acute exercise and eye closure has a synergistic effect on alpha activity

**DOI:** 10.1038/s41598-021-99783-y

**Published:** 2021-10-12

**Authors:** Takaaki Komiyama, Ryoma Goya, Chisa Aoyama, Yusuke Yokota, Yasushi Naruse, Satoshi Shimegi

**Affiliations:** 1 Center for Education in Liberal Arts and Sciences, Osaka University, 1-16 Machikaneyama, Toyonaka, Osaka 560-0043 Japan; 2 Graduate School of Frontier of Biosciences, Osaka University, Toyonaka, Osaka Japan; 3 Graduate School of Medicine, Osaka University, Toyonaka, Osaka Japan; 4 Center for Information and Neural Networks (CiNet), National Institute of Information and Communications Technology, Osaka University, Kobe, Japan

**Keywords:** Neuroscience, Physiology

## Abstract

Acute aerobic exercise increases the brain cortical activity in alpha frequency. Eye closure also increases alpha activity. However, whether the two have an additive or a synergistic effect on alpha activity has never been explored. This study observed electroencephalography (EEG) from fifteen participants seated on the cycle ergometer before, during, and after a cycling exercise with the eyes open and with them closed. Exercise intensity was set to a target heart rate (120–130 bpm), corresponding to light-to-moderate intensity exercise. Each epoch was 6 min and the last 4 min (eyes closed in the first 2 min and eyes open in the second 2 min) were analyzed. The EEG power spectrum densities were calculated for alpha frequency band activity (8–13 Hz). At rest, alpha activity was significantly greater with the eyes closed than open. Exercise significantly increased alpha activity in both eye conditions. More importantly, in the occipital site, the alpha-increasing effect of their combination was significantly greater than the sum of the effect of each, showing a synergistic effect. We concluded that acute light-to-moderate intensity exercise with the eyes closed has a synergistic effect on alpha activity.

## Introduction

Behavioral state changes brain cortical activity, which can be recorded by electroencephalography. Cortical activity has rhythmicity represented as theta, alpha, and beta frequency bands. Acute aerobic exercise seems to influence cortical activity in these frequency band^1,2^. Among them, previous studies have reported that acute aerobic exercise temporally increases alpha activity in anterior sites of brain^3,4^. Moreover, the increase in alpha activity is accompanied by an improvement in mood after acute exercise^4–7^. Thus, acute aerobic exercise potentially has beneficial effect for mood state through increase in alpha activity.

The effect of exercise on alpha activity depends on the exercise condition such as intensity. Several previous studies have shown that alpha activity increases from moderate intensity to vigorous intensity in well-trained participants, but it decreased at high intensity^8–10^. Although the obvious mechanism is still unknown, the increase in alpha activity during exercise associates with neuromodulators^5,7,11^. In fact, it has been reported that positive correlation was observed between increase in alpha activity and noradrenalin level after exercise^11^. Given that noradrenalin level notably increases at moderate intensity exercise correspond to lactate threshold, near moderate-exercise intensity is likely an efficient means for increasing alpha activity.

Similar to acute aerobic exercise, it is well known that eye closure increases alpha activity^12^. The increased alpha activity during eye closure is dominantly observed at occipital regions^12,13^, and the alternation in alpha activity involved eyes behavior (open or close) seems to arise as a consequence of changing external inputs to the inhibitory neurons in the cortex^14^. Therefore, the mechanism of increase in alpha activity is likely to be different pathway between acute aerobic exercise and eye closure. However, since the effects of these factors on alpha activity have been investigated individually so far, it remains unclear whether the mechanisms of alpha activity induced by exercise and eye closure are common or different, and if they are different, whether they are independent or interact with each other. An important clue to clarify these points is whether the effects of combining the two factors are additive or synergistic. Several studies have examined the effect of exercise with eye closure on brain cortical activity^7,9,11,15,16^, but the differences between the eyes closed and open during exercise on alpha activity have been never directly compared. Therefore, the purpose of this study was to clarify whether eye closure elicits an increase in alpha activity during exercise, and how the effects of exercise and eye closure interact with each other.

## Results

For heart rate (HR) and the power spectrum density (PSD) of brain cortical activity (alpha, beta, theta frequency band) at each brain region (occipital, parietal, frontal), we performed two-way repeated-measures ANOVA [Eye condition (EC: eyes closed, and EO: eyes open) × Physical condition (Rest, Exercise, and Recovery), and then post-hoc test by using a multiple comparison with the Bonferroni correction when main or interaction effects were observed.

### HR and RPE

Table 1 shows the average HR throughout the actual experiments. There was a significant main effect of Physical condition (F_1.19, 16.64_ = 224.44, *p* < 0.001), but not interaction. HR was significantly higher during Exercise (8–12 min) compared to Rest (2–6 min) (*p* < 0.001). The HR in Recovery (14–18 min) was lower than Exercise (*p* < 0.001) but higher than Rest (*p* < 0.001). On the other hand, there was not a significant main effect of Eye condition (F_1, 14_ = 3.55, *p* = 0.08), neither EO nor EC significantly affected the HR at any physical state. These results indicate that the HR change caused by exercise reached a stable state within 2 min after the exercise began and recovered to the rest state within 2 min after it ended, and the physiological state during or after exercise were equally between eyes condition.

**Table 1:** Average HR during the experiments.

Physical condition	Rest		Exercise		Recovery	
Eye condition	EC	EO	EC	EO	EC	EO
Time	(2–4 min)	(4–6 min)	(8–10 min)	(10–12 min)	(14–16 min)	(16–18 min)
HR, beats min^−1^	75.3 ± 9.9	76.4 ± 9.8	122.0 ± 3.6^a^	123.7 ± 4.5^a^	84.1 ± 11.3^a,b^	84.7 ± 10.6^a,b^

Values are mean ± SD.

*HR* heart rate, *EC* eye closed, *EO* eye open.

^a^p < 0.01, vs. rest (2–6 min).

^b^p < 0.01 vs exercise (8–12 min).

The RPE (rating of perceived exertion) during exercise was 12.3 ± 0.9 on average, and the range within all participants was 10–13. This indicates that the exercise workload corresponded to light-to-moderate intensity for all participants.

### Power spectrum density (PSD)

#### Alpha frequency band

**Figure 1:**
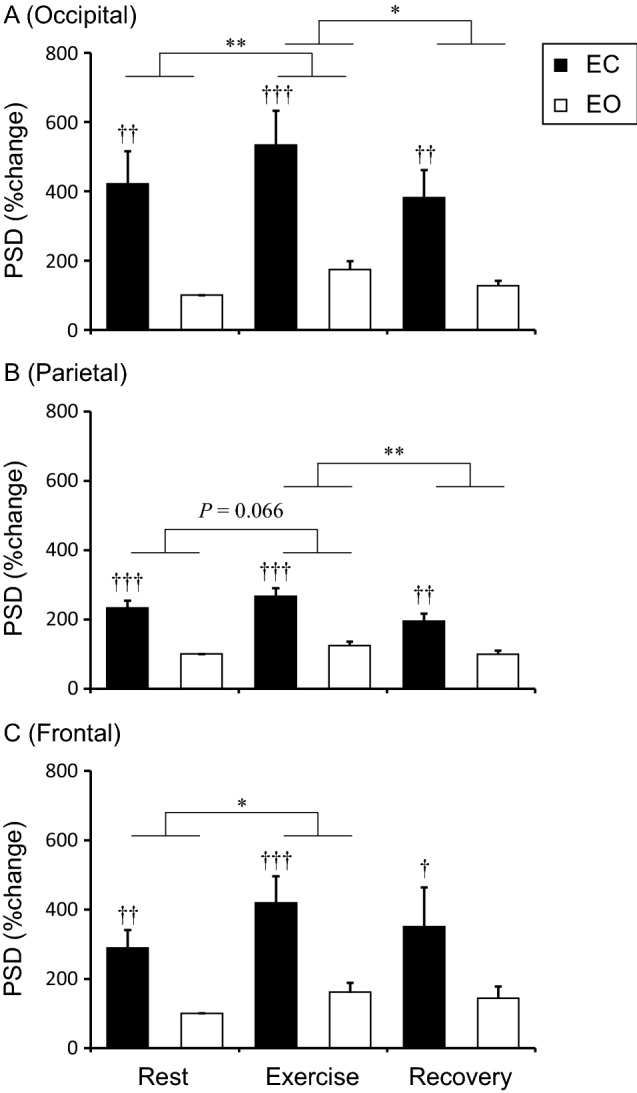
Alpha power spectrum densities in occipital **(A)**, parietal **(B)**, and frontal **(C)** site at the rest, exercise, and recovery periods. The values indicate the percentage change from Rest + EO and are shown as the mean ± standard error (N = 15). *p < 0.05, **p < 0.01. †*p* < 0.05, ††*p* < 0.01, †††*p* < 0.001 vs EO. *PSD* power spectrum densities.

Figure 1 shows α-PSD at rest, during exercise, and after exercise as the rate of change from Rest-EO. In the occipital (Fig. 1A) and parietal (Fig. 1B) sites, the analysis of α-PSD revealed a significant main effect of Eye (occipital, F_1,14_ = 13.78, *p* < 0.01; parietal, F_1,14_ = 40.22, *p* < 0.001) and Physical condition (occipital, F_1.35, 18.85_ = 11.77, *p* < 0.01; parietal, F_2, 28_ = 9.56, *p* < 0.01) and interaction (occipital, F_2,28_ = 4.63, *p* = 0.018; parietal, F_2, 28_ = 3.59, *p* < 0.01), respectively. In both regions, α-PSD was higher in EC than EO at Rest (both, *p* < 0.01), Exercise (both, *p* < 0.001), and Recovery (both, *p* < 0.01), suggesting that eye closure increased α-PSD regardless of the physical state. In the occipital region, exercise also significantly increased α-PSD in both eye conditions (Rest vs. Exercise, *p* < 0.01). In the parietal site, exercise tended to increase α-PSD regardless of eye condition (Rest vs. Exercise, *p* = 0.066). However, the increment effect disappeared in Recovery in occipital (Exercise vs. Recovery, *p* = 0.011) and parietal site (Exercise vs. Recovery, *p* < 0.01). In the frontal site, a significant interaction was not observed. There was a significant main effect of Eye condition (F_1, 14_ = 12.90, *p* < 0.01), in which eye closure significantly increased α-PSD at any Physical conditions (Rest, *p* < 0.01; Exercise, *p* < 0.001; Recovery, *p* = 0.028). Although the main effect of Physical condition was not significant (F_1.45, 20.33_ = 2.69, *p* = 0.085), exercise significantly increased α-PSD regardless of eye condition (Rest vs. Exercise, *p* = 0.022).

We performed the additional experiment with the eye conditions in reverse order and observed similar results as described above (Fig S1A). These results indicate that increase in alpha activity during exercise with eyes closed was not caused by the particular order of eye conditions from closed to open.

#### Beta frequency band

**Figure 2:**
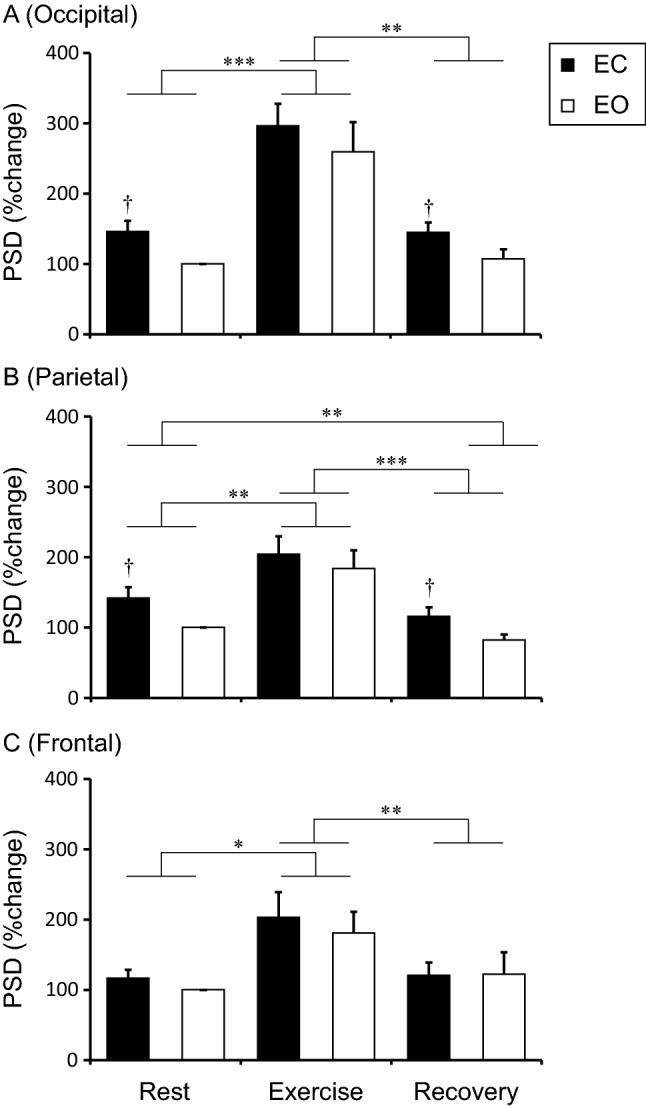
Beta power spectrum densities in occipital (**A**), parietal (**B**), and frontal (**C**) site at the rest, exercise, and recovery periods. The values indicate the percentage change from Rest + EO and are shown as the mean ± standard error (N = 15). *p < 0.05, **p < 0.01, ***p < 0.001. †*p* < 0.05 vs EO. *PSD* power spectrum densities.

Figure 2 shows β-PSD at rest, during exercise, and after exercise as the rate of change from Rest-EO. A significant interaction between the Eye condition and the Physical condition was not observed for any cortical regions. The main effect of the Physical condition on β-PSD was significant for all recording sites (occipital, F_1.18, 16.57_ = 20.46, *p* < 0.01; parietal, F_1.15, 16.10_ = 19.25, *p* < 0.001; frontal, F_1.33, 18.67_ = 7.47, *p* < 0.01). In all the sites, β-PSD showed a significant increase during Exercise compared to Rest that was independent of the eye condition (all *p* < 0.05), but the effect disappeared in Recovery (Exercise vs. Recovery, all *p* < 0.01). For occipital and parietal sites, there was a significant main effect of Eye condition (occipital, F_1, 14_ = 8.77, *p* < 0.05; parietal, F_1, 14_ = 5.64, *p* < 0.05), but not frontal site (F_1, 14_ = 1.50, *p* = 0.24). Eye closure increased β-PSD at Rest and Recovery (all *p* < 0.05).

#### Theta frequency band

**Figure 3:**
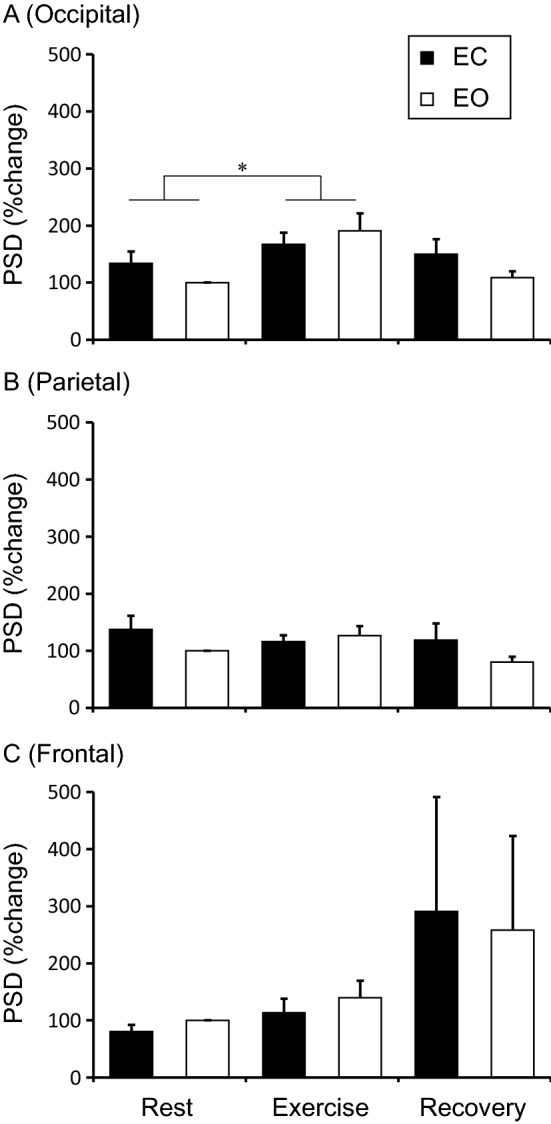
Theta power spectrum densities in occipital **(A)**, parietal **(B)**, and frontal **(C)** site at the rest, exercise, and recovery periods. The values indicate percentage change from Rest + EO and are shown as the mean ± standard error (N = 15). *p < 0.05. *PSD* power spectrum densities.

Figure 3 shows θ-PSD at rest, during exercise, and after exercise as the rate of change from Rest-EO. In all the regions, a significant interaction and main effect of Eye condition were not observed. There was a significant main effect of Physical condition only in the occipital site (F_2, 28_ = 4.10, *p* < 0.05). Exercise increased θ-PSD regardless of eye condition (Rest vs. Exercise, *p* < 0.05).

#### Additive vs. synergistic effects for alpha activity

**Figure 4:**
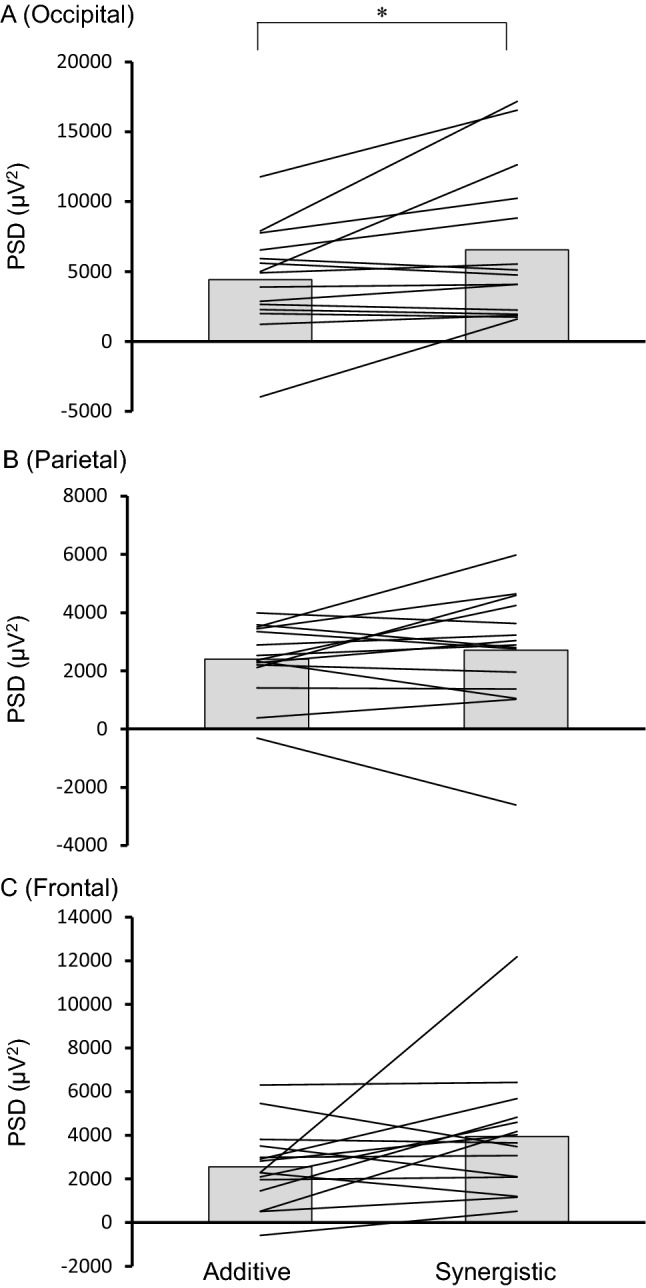
An index of additive and synergistic effects for alpha activity in occipital **(A)**, parietal **(B)** and frontal **(C)** site. The bars indicate means (N = 15). The solid lines indicate individual data (N = 15). *p < 0.05. *PSD* power spectrum densities.

We found that both exercise and eye closure increase α-PSD and that the effect of their combination was greater than the effect of each in some region. Therefore, we examined whether the combined effect was additive or synergistic for all the region (Fig. 4). For occipital region, we found the synergistic effect was significantly larger than the additive effect (paired T-test, t_14_ = -2.57, *p* < 0.05). A significant difference was not observed between additive or synergistic effects in other brain regions (paired T-test, t_14_ = -0.87, *p* = 0.39). These results indicate that exercise with the eyes closed increases alpha activity synergistically only at the occipital region.

The same results were also reproduced in the additional experiments performed with the reversed order of the eye conditions (Fig S1B), showing that the synergistic effect was significantly larger than the additive effect (paired T-test, t_14_ = -2.67, *p* < 0.05).

## Discussion

This study aimed to determine whether eye closure elicits an increase in alpha activity during exercise, and how the effects of exercise and eye closure interact with each other. The main findings of this study were: (1) short, light-to-moderate intensity exercise increased the alpha and beta activity; and (2) the increase in alpha activity during exercise was higher with the eyes closed than with the eyes open, and the effect was synergistic only at the occipital region. These findings suggest that short, light-to-moderate intensity exercise with the eyes closed elicits a synergistic effect for alpha activity.

Short, light-to-moderate intensity exercise temporarily increased alpha and beta activity at almost brain regions in the present study. EEG measurement during exercise often includes artifacts due to upper body and head movements. If the increase in alpha and beta theta activity during exercise resulted from artifacts such as noise generated by the physical movements, the increment should be observed at all the recording sites. However, the increase in theta activity was only observed at occipital site, implying that the exercise effects on alpha and beta activity were not artifacts.

It is widely accepted that acute exercise alters brain cortical activity^2^, and the effect of exercise on the activity seems to depend on exercise intensity and duration^8–10,15,17,18^. It has been reported that both alpha and beta activity increase as exercise increases from low to moderate intensity, but then decreases as the exercise reaches high intensity such as exhaustion^8–10^. Also, short or moderate durations of exercise increase brain cortical activity (from theta to beta frequency), but longer durations cause the increase to disappear^17,18^. Thus, exercise intensity/duration influences the effects of exercise on brain cortical activity, and a physiological load has been recognized as an important determinant of exercise effects. Currently, there is no consensus on the relationship between exercise intensity/duration and cortical activity, but those exercise conditions may have requirements to increase cortical activity. In this study, a significant increase in alpha and beta activity was observed with light-to-moderate intensity exercise even in a short period of minutes. Similar results to this study were obtained in previous studies of short-term moderate-intensity exercise^15,19^. Therefore, it can be considered that the short but light-to-moderate exercise conditions adopted in this study met some requirements for increasing cerebral cortical activity. Several previous studies have suggested that increased alpha and beta activity is associated with physiological responses such as lactate or noradrenaline secretion^11,15,17^. This study did not measure those physiological responses, but those physiological changes may have been associated with changes in cortical activity.

We measured EEG after exercise to examine how long the effect of exercise lasted. Several studies reported that an exercise-induced increase in brain cortical activity is sustained after cessation of the exercise^2,9,20^. Contrary to this finding, the present study observed that the alpha and beta activity quickly returned to baseline immediately after the exercise was terminated. The discrepancy between the studies seems to be explained by the exercise load, such as intensity and duration. A previous study reported that alpha and beta activity increased immediately after moderate-intensity exercise (50% VO_2max_) for 30 min^9^. A second showed that the increase in alpha activity following exercise was longer at a higher exercise workload^20^. In the present study, we employed light-to-moderate intensity exercise (HR, 120–130 bpm) for 6 min. This exercise load was relatively low compared to the aforementioned studies^9,20^. Thus, the short, light-to-moderate intensity exercise used in the present study was insufficient for sustaining the increase in alpha and beta activity after the exercise. Further study needs to investigate what exercise condition (intensity, duration, and/or volume) sustains the beneficial effects on brain cortical activity.

We directly compared alpha activity during exercise with the eyes open and closed and also examined whether exercise with the eyes closed elicits a synergistic effect on alpha activity. Indeed, the combination of exercise and eye closure increased alpha activity synergistically at the occipital region. This is the first study to directly compare differences between closed and open eyes during exercise on brain cortical activity. The mechanism underlying the synergistic effect is unclear. However, the synergetic effect does suggest that the mechanisms of the exercise effect and eye closure effect on alpha activity are not independent but interact with each other. Each mechanism has been investigated individually. It has been suggested that several neuromodulators such as noradrenalin or serotonin associate with alpha activity^21,22^. In addition, one study found a positive correlation of the plasma noradrenaline level with alpha activity after exercise, implying the noradrenergic system is associated with the increase in alpha activity^11^. Similarly, other studies showed a relationship between the serotonin level and alpha activity during cycling exercise^5,7^. Since these noradrenergic and serotonergic systems are activated by exercise and regulate brain activity extensively, the exercise-induced increase in global alpha activity may be mediated by these two neuromodulators^2,6,11,15,23^. To support this notion, increase in alpha activity was observed during exercise at global brain regions in this study. On the other hand, eye closure has been reported to increase alpha activity via a change in external inputs to inhibitory neurons in the cortex^14^. Therefore, the mechanism of increase in alpha activity is likely to be a different pathway between exercise and eye closure. Not only that, eye closure seems to increase alpha activity in relation to not only a reduction of visual input but also to the closing of the eyelids themselves. Several studies found that alpha activity is increased by closing the eyelids even in darkness^24,25^. These findings suggest that closed eyes act as a behavioral cue to switch to a brain mode where the alpha activity is dominant^24,26–29^. In fact, fMRI studies discovered that closing the eyelids activates specific brain areas and networks relating to alpha activity^28,30^. Given that the neuromodulators secreted by exercise modulate various networks in the brain extensively, an alpha generator activated during eye closure may be enhanced by the exercise-induced neuromodulators. If this possibility is true, the synergistic effect on alpha activity should be observed more remarkably at the occipital region, because eyes closure alone increases alpha activity dominantly there^13^. In accordance, we found the synergistic effect on alpha activity was most remarkable at the occipital region. Future work should consider which networks are involved in generating alpha activity and how those networks are modulated by exercise and eye closure.

We cannot rule out the possibility that the relative physiological load during exercise was different among participants. In the present study, the exercise intensity was set by a fixed target HR (120–130 bpm) across participants according to their age, where an HR of 120–130 bpm corresponds to light-to-moderate intensity for most participants (approximately 60–65% HR_max_)^31^. However, the exercise intensity may have been different for some participants since the relation between HR and physiological load varies among participants depending on their fitness level. This could be a limitation when extending this study to different exercise conditions. Further studies should use a constant workload (e.g. % VO_2 peak_) across participants.

This is the first study that directly compared the effects of acute exercise on alpha activity with the eyes opened and closed. In summary, both short, light-to-moderate intensity exercise and eye closure increased alpha activity, and their combination did so synergistically at only the occipital region. These findings suggest that the mechanisms of the exercise effect and eye closure effect on alpha activity are not independent but interact with each other.

## Methods

### Participants and ethical approval

Fifteen healthy young adults (mean ± SD: age = 21.0 ± 2.3 years, height = 1.67 ± 0.08 m, body mass = 61.1 ± 9.8 kg; 2 females) were recruited in this study. In additional experiment, we recruited fourteen healthy young adults (mean ± SD: age = 21.9 ± 3.2 years, height = 1.69 ± 0.10 m, body mass = 64.6 ± 11.4 kg; 3 females). They had no history or symptoms of cardiovascular, neurological, or respiratory diseases. All the participants were in good shape on an experimental day. This study was approved by the Osaka University Human Ethics Committee (16,207). The study conformed to the standards set by the latest revision of the Declaration of Helsinki, except for registration in a database. All the participants provided written informed consent.

### Experimental procedure

All experiments were conducted in a dim room. As a preliminary test, each participant briefly cycled on a cycle ergometer (Aerobike 75XLIII; Combi, Tokyo, Japan) while the heart rate (HR) was monitored to determine the exercise workload for the actual test. After warming-up at 10 watts for 1 min, the exercise workload was increased until reaching the target HR (120–130 bpm). Then the participants cycled for 3 min at the determined exercise workload to confirm whether the target HR was maintained. After the preliminary test, the participants were equipped for EEG measurements while they rested.

**Figure 5:**
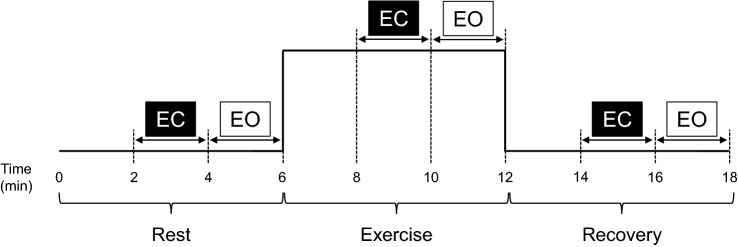
The experimental protocol. *EC* eyes closed, *EO* eyes opened.

As for the actual experiments, the EEG was measured before, during, and after the exercise with eyes opened and closed (Fig. 5). At the beginning of the experiments, the participants were seated on the cycle ergometer at rest for 6 min (Rest). We instructed the participants to remain quiet and avoid any movement. Then they performed the cycling exercise at the target HR for 6 min (Exercise). They were asked to maintain a cycling cadence of 60 rpm and to cycle with minimal upper body and head movements. After completing the exercise, the participants remained seated on the cycle ergometer at rest for 6 min (Recovery). At the last 4 min of each epoch, the participants kept the eyes closed for 2 min (EC) and open for the remaining 2 min (EO). The onset of EC and EO was orally instructed by the experimenter. During EO, the participants were asked to look at a small cross-fixation on the wall in front of them^26^. All participants were relaxed throughout the experiments and made no comment about fatigue, anxiety, or tension after the experiment.

### Additional experiment

The experimental procedure, which sets the eye condition in a fixed order of closing the eyes and then opening the eyes, can have a confounding effect of order or time. Therefore, to confirm this point, we conducted an additional experiment to reverse the eye condition at each stage, where the eye condition order was EC, EO, EC. There were no changes to the experimental conditions other than the order of eye conditions.

### HR and RPE measurements

HR was monitored using a HR monitor (V800; Polar Electro Oy, Kempele, Finland) throughout the experiment. The rating of perceived exertion (RPE, 6–20 scale) was measured during the exercise^32^. The RPE consists of the 15-grade ranging scale from "6" to "20", in which, for example, the grades "7", "9", "11", "13", "15", "17", and "19" correspond to "Very very light", "Very light", "Fairly light", "Somewhat hard", "Hard", "Very hard", and "Very very hard", respectively.

### EEG recordings and analysis

We measured EEG signals using an 8-channel wireless EEG system (Polymate Mini AP108; Miyuki Giken, Tokyo, Japan)^33^. Six dry electrodes were placed on the bilateral hemispheres (Occipital: O1, O2, Parietal: P3, P4, Frontal: F3, F4) according to the 10–20 system^34^ using a stretchable electrode cap (g.GAMMAcap2; Miyuki Giken, Tokyo, Japan). A paired mastoid was used as a reference site, which was cleaned and abraded before the electrodes were placed. The impedance of all electrodes was below 300 kΩ. The EEG data were sampled at 500 Hz.

EEG analyses were performed using MATLAB (Mathworks, Inc., Natick, MA, USA). We excluded the first 2 min of each stage (Rest, Exercise, and Recovery) from the analysis, since the physiological response may not have achieved steady state in this time. For each 2 min EEG recording (EC and EO in Rest, Exercise, and Recovery), the first 80 s and last 10 s were eliminated to ensure the data were not contaminated by state transitions such as shifting condition (start of the exercise or eye condition) and to get instructions from the experimenter, respectively. According to a previous study^19^, the 30 s of data from 80 to 110 s of each epoch were processed by a digital band-pass filter (Butterworth; 1.5–30 Hz). Automatic rejection of artifacts such as eye blink was applied with an amplitude criterion (amplitudes outside -100 μv to 100 μv), and the data sequence 100 ms before and after the event was removed. A power spectrum was calculated from the EEG signal using a fast Fourier transformation for each epoch. Power spectrum densities (PSD) in the theta (4–7 Hz), alpha (8–13 Hz), and beta (13–30 Hz) frequency bands were quantified from the average power spectrum in their frequency bands for the occipital (average of O1 and O2), parietal (average of P3 and P4) and frontal sites (average of F3 and F2), respectively. We analyzed the theta and beta frequency bands to ensure whether the interactive effect of exercise and eye closure specifically appears at alpha frequency band.

### Data and statistical analysis

The HR was averaged every epoch (EC and EO in Rest, Exercise, and Recovery). The averaged PSD was expressed as a relative change from Rest-EO (as 100%) to reduce inter-individual variability as suggested by previous studies^19,35^. Rest-EO was used as the baseline for relative evaluation to compare the effects of exercise and eye closure individually and in combination on the same basis, because eye-closed resting state is adopted as baseline may obscure the significant difference on alpha activity^1^. A two-way repeated-measures ANOVA [Eye condition (EC and EO) × Physical condition (Rest, Exercise, and Recovery)] was performed for HR and each PSD of brain region (occipital, parietal, frontal) or frequency band (theta, alpha, and beta). The degree of freedom was corrected using the Greenhouse–Geisser correction when the assumption of sphericity was violated. Where main or interaction effects were observed, post-hoc test was conducted using a multiple comparison with the Bonferroni correction. We examined whether the effect of exercise and eye closure interact with each other was additive or synergistic by using α-PSD. The additive effect shows sum of each effect of eye closure and exercise. Thus, the additive effect was estimated by the following equation: (Rest EC – Rest EO) + (Exercise EO – Rest EO). On the other hands, if the increase shows over the sum of each effect of eye closure and exercise, it could be regarded as the synergistic effect. Therefore, the synergistic effect was calculated as the following equation: Exercise EC – Rest EO. Then, we determined which the combined effect is additive or synergistic by comparison between the calculation results using paired T-test. All EEG data are expressed as means ± SE. The significance level was set at *p* < 0.05.

## Supplementary Information

Supplementary Figure S1.

Supplementary Table S1.

## Data Availability

The datasets generated during and/or analyzed during the current study are available from the corresponding author on reasonable request.
