# Supplementary material for: The combination of acute exercise and eye closure has a synergistic effect on alpha activity
Source: Sci Rep. 2021 Oct 12;11:20186. doi: 10.1038/s41598-021-99783-y (PMC8511023; doi:10.1038/s41598-021-99783-y)
Supplement: Supplementary file 1 — Supplementary Figure S1. [file 41598_2021_99783_MOESM1_ESM.pdf]

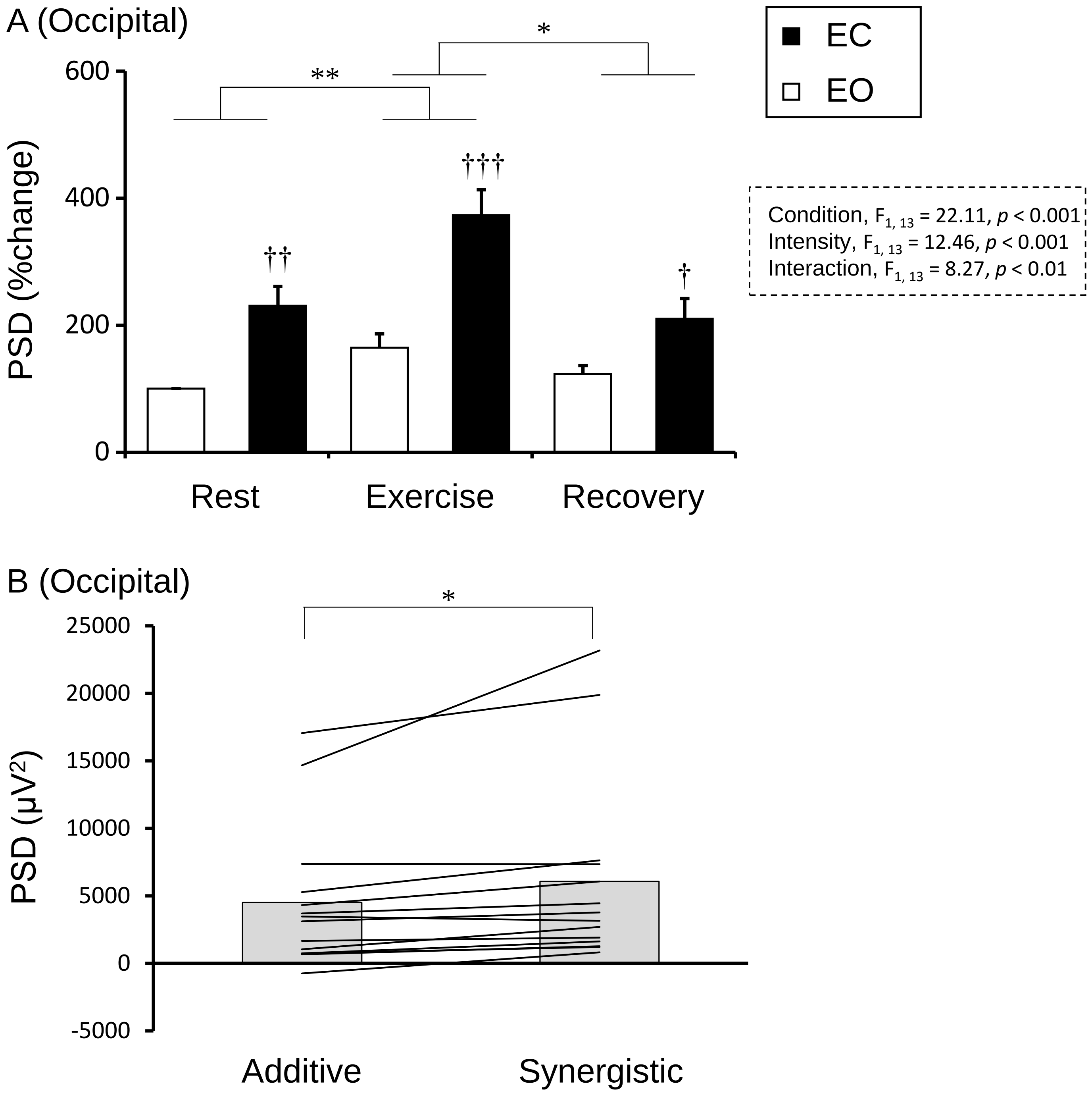

Figure S1: Additional experiment. Alpha power spectrum densities in occipital (A) site at the rest, exercise, and recovery periods. The values indicate the percentage change from Rest + EO and are shown as the mean ± standard error (N = 14). An index of additive and synergistic effects for alpha activity in occipital (B). The bars indicate means (N = 14). The solid lines indicate individual data (N = 14). *p < 0.05, **p < 0.01. †p < 0.05, ††p < 0.01, †††p < 0.001 vs EO. PSD, power spectrum densities.
