# Supplementary material for: The combination of acute exercise and eye closure has a synergistic effect on alpha activity
Source: Sci Rep. 2021 Oct 12;11:20186. doi: 10.1038/s41598-021-99783-y (PMC8511023; doi:10.1038/s41598-021-99783-y)
Supplement: Supplementary file 2 — Supplementary Table S1. [file 41598_2021_99783_MOESM2_ESM.pdf]

Table S1. Alpha acitivity in occipital site during the experiments.

Subjects	Rest		Exercise		Recovery	
	EC	EO	EC	EO	EC	EO
001	568	100	734	195	540	300
002	167	100	207	155	141	117
003	199	100	227	95	157	76
004	192	100	276	123	177	104
005	267	100	552	141	105	168
006	150	100	428	180	84	125
007	946	100	1258	354	879	101
008	111	100	111	61	77	76
009	403	100	546	115	433	125
010	1523	100	1548	385	1189	160
011	631	100	601	150	655	138
012	217	100	493	259	388	74
013	320	100	357	75	315	97
014	372	100	446	236	309	158
015	260	100	219	80	276	92

The averaged PSD was expressed as a relative change from Rest-EO (as 100%). EC, eye closed, EO, eye open.
